# Physical activity during life course and bone mass: a systematic review of methods and findings from cohort studies with young adults

**DOI:** 10.1186/1471-2474-14-77

**Published:** 2013-03-04

**Authors:** Renata M Bielemann, Jeovany Martinez-Mesa, Denise Petrucci Gigante

**Affiliations:** 1 Post-Graduate Program in Epidemiology, Federal University of Pelotas, Pelotas, Brazil; 2 Department of Nutrition, Federal University of Pelotas, Pelotas, Brazil

## Abstract

**Background:**

The purpose of this paper was to review the literature of the cohort studies which evaluated the association between physical activity during the life course and bone mineral content or density in young adults.

**Methods:**

Prospective cohort studies with bone mineral density or content measured in the whole body, lumbar spine and femoral neck by dual energy x-ray absorptiometry as outcome and physical activity as exposure were searched. Two independent reviewers selected studies retrieved from electronic databases (Medline, Lilacs, Web of Science and Scielo) and reviewed references of all selected full text articles. Downs & Black criterion was used in the quality assessment of these studies.

**Results:**

Nineteen manuscripts met inclusion criteria. Lumbar spine was the skeletal site most studied (n = 15). Different questionnaires were used for physical activity evaluation. Peak strain score was also used to evaluate physical activity in 5 manuscripts. Lack of statistical power calculation was the main problem found in the quality assessment. Positive associations between physical activity and bone mass were found more in males than in females; in weight bearing anatomical sites (lumbar spine and femoral neck) than in total body and when physical activity measurements were done from adolescence to adulthood – than when evaluated in only one period. Physical activity during growth period was associated with greater bone mass in males. It was not possible to conduct pooled analyses due to the heterogeneity of the studies, considering mainly the different instruments used for physical activity measurements.

**Conclusions:**

Physical activity seems to be important for bone mass in all periods of life, but especially the growth period should be taking into account due to its important direct effect on bone mass and its influence in physical activity practice in later life. Low participation in peak strain activities may also explain the lower number of associations found in females.

## Background

Currently osteoporosis, which is characterized by a reduction in bone mass [1], is a worldwide health problem with great social and financial impact on society [2]. Osteoporosis increases the risk of fracture due to low bone mass and deterioration of its structure which causes bone fragility [1].

There is some evidence to suggest that the risk of osteoporosis and its related-problems may be reduced by maximizing the accrual of peak bone mass in the first few decades of life [3]. In addition, the bone mass present at a given time in life is determined by the factors that influence the gain, maintenance or bone loss across the lifespan, including modifiable and lifestyle factors.

Physical activity is a relevant factor to prevent or treat osteoporosis for its capacity to increase or reduce bone loss due to modifications in bone structure and geometry caused by mechanical loads applied from physical activity to bones that stimulate osteogenic responses [4]. Moreover, physical activity also improves strength, flexibility, coordination, balance, reaction time and endurance. However, there are uncertainties about the type, the intensity, the duration, and the frequency of the physical activities that are optimal for an increase in bone mineral density [5].

Evidence supporting the role of physical activity in bone health has accumulated from cross sectional, cohort and intervention studies. Cross-sectional studies have limitation of temporality, because such studies often have difficult determining the time order of events. On the other hand, randomized-controlled trials show large dropout rates and need long periods of time to achieve measurable changes in bone mass [6]. Furthermore, RCTs are carried out using specific types of activities with different volumes, duration and intensities, which do not represent physical activity general populations. Thereat, the knowledge from longitudinal observational studies (cohorts) is relevant, in which it is possible to evaluate the effect of physical activity on bone mass at a given time in life or across the lifespan, when there are only a few if any RCTs. Moreover, observational studies allow for different kinds of the same exposure to be analyzed in the same sample, making the comparison between effects of different activities easier.

Therefore, the purpose of this study was to review the literature about cohort studies which evaluated the longitudinal association between physical activity during the life course (childhood, adolescence and adulthood) and bone mineral content or density in young adults, describing their samples, methods, quality, differences, findings and fragilities.

## Methods

### Search strategy

The literature search was conducted in the databases Pubmed, LILACS, Scielo and Web of Science. The search was performed by a single author and occurred up to May 2012 without date limits or language restrictions. Three command groups were employed to find articles. In the first group, we included the terms related to bone mineral density or content (bone density; bone mineral density; bone mass; bone mineral content; bone content). In the second one the terms related to physical activity were entered (physical activity; motor activity; inactivity; sedentarism; sedentary; sports; exercise). In the third group, we added the terms to restrict the study design (longitudinal; cohort; prospective; follow-up). Within each group, we used the Boolean operator ‘OR’ and between the groups we used the Boolean operator ‘AND’. In the Pubmed database we restricted the search for studies performed with adults (19–44 years), whereas in the other ones we added a fourth group of commands related to age group (adults, young adults, adulthood).

### Selection of studies

A database with the search results was generated, excluding duplicate references, totaling 750 articles. The selection of articles included in the final review was performed independently by two reviewers (RMB and JMM), based on inclusion and exclusion criteria previously defined. In the case of disagreement, the selection was evaluated by a third reviewer (DPG). Initially, each reviewer selected the titles for articles of interest. The second step consisted of the examination of abstracts from those papers previously selected. Then, we proceeded to search the full text. The references of all selected full text articles were also reviewed.

### Eligibility criteria

Criteria used to identify the manuscripts were regarding subjects, study design and measurement of outcomes. Concerning the subjects, studies should be conducted in healthy adult subjects with age from 20 to 40 years (or average in this interval) and not specifically athletes. The age was limited up to 40 years though the maintenance phase of bone mass occurs during young to middle adulthood [1], decreases on bone mass occur at earlier ages, mainly in women, due to premenopausal or menopausal periods. Another criterion was that the studies should evaluate bone mass using the method of dual energy x-ray absorptiometry (DXA) in at least one out of these three sites: total body, lumbar spine and femoral neck. The choice of this method was due to the evidence shown by the literature that DXA is the main method for evaluation of bone mineral density. Besides, it is the gold standard to diagnose osteoporosis [1,7-9]. Regarding the study design, we included only cohort studies, which performed at least one longitudinal analysis between physical activity and outcomes.

### Exclusion criteria

We excluded studies in which the sample was made up by subjects with diseases which are known to affect the bone metabolism (i.e. lupus erythematosus, cerebral palsy, cancer, etc.) and those that used other methods to measure bone mass. Cohort studies which performed only cross-sectional analysis between main exposure and outcomes were also excluded.

### Quality assessment

The evaluation of the quality of evidence was also performed independently by two authors (RMB and JMM). The disagreements were discussed between the two authors and the final decision was made by consensus between the two examiners. The instrument proposed by Downs and Black [10] was used to assess the quality of studies. These authors devised an instrument consisting of 27 questions that evaluate reporting, external validity, internal validity (bias and confounding), and statistical power. In items 4, 14, and 15, “intervention” was interpreted as “exposure,” and in no. 19 “compliance with the intervention” was replaced by “avoidance of misclassification error of the exposure”. Since the instrument was originally conceived for the evaluation of clinical trials, items applicable specifically to this study design (8, 13, 23, and 24) were not considered. All questions received scores 0 or 1, with the exception of question 5, which ranged from 0 to 2, depending on whether the statistical power of the survey was explicitly stated in the article as being at least 80%. Thus, the maximum score achievable by an article was 24 points. This manuscript was written according to current recommendations of Preferred Reporting Items for Systematic Reviews and Meta-Analyses (PRISMA) Statement [11].

## Results

### Description of the studies

**Figure 1:**
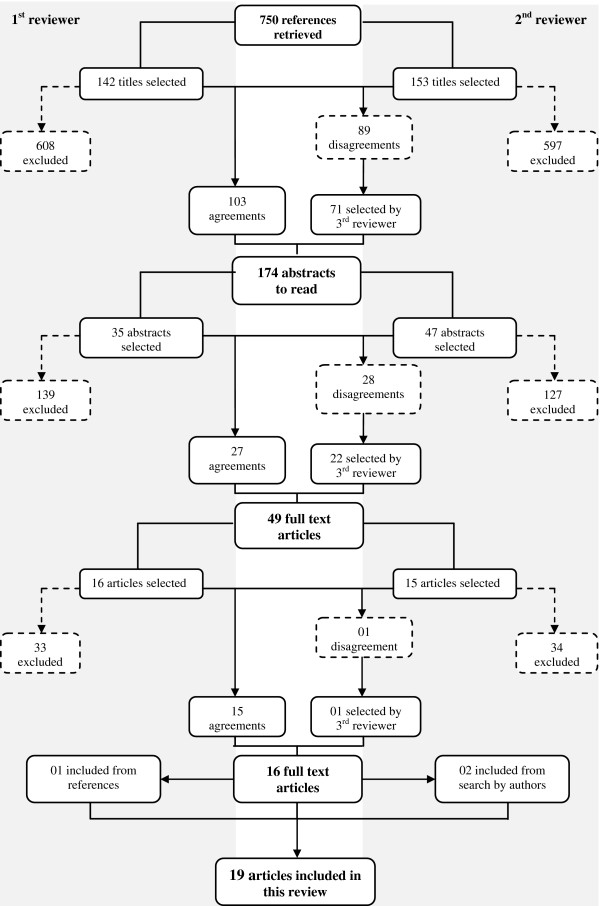
Flow diagram of study selection.

Figure 1 shows the study selection flowchart. Out of the 750 references initially located, 576 of the potential articles were excluded in the first step as the focus was not on PA; were conducted in unhealthy subjects, children, adolescents, postmenopausal women, elderly or athletes; or were cross-sectional studies. From the 174 papers with abstracts assessed, 49 were selected for reading the full text, based on the inclusion criteria. Out of these, 33 were excluded. The main reasons were the study design and age of subjects included in the sample [12-30]. Other reasons for exclusion were the method for evaluation of bone mass [31-35], as studies did not evaluate the effect of physical activity on bone mass [36-41] or were a review of findings showed in other articles conducted with the same sample [42]. The reference lists of all selected papers were examined to detect other publications eligible for this review. In this process we identified one article which was not found before [43]. In the end, two other studies were found by search using the all author’s names of included manuscripts followed of terms related to bone mineral density or content previously described. In total, 19 articles were selected for this review.

The description of these studies is shown in Table 1. The most part of studies have been published in the last 12 years. We only found studies carried out in high income country. Out of these, the majority (*n* = 14) was performed in Europe. Concerning sample size, few studies (*n* = 6) had more than 200 subjects. Seventeen studies were carried out with females, whereas 11 were performed with males. Only three of these cohort studies did not evaluate the effect of physical activity during childhood or adolescence on bone mass. Three studies performed analysis between physical activity during adulthood and bone mass. Few studies used bone mineral content as outcome (*n* = 6), whereas bone mineral density was not evaluated only in three out of these 19 studies. Lumbar spine was the skeletal site most studied (*n* = 15). Femoral neck was evaluated by 13 studies, whereas association between physical activity and total body bone mineral density or content was showed in only five articles.

**Table 1:** Description of studies included in the present review

**Study characteristics**	**N**	**%**
**Year of publication**		
Up to 2000	5	26.3
2001 – 2012	14	73.7
**Continent**		
North America	4	21.0
Europe	14	73.7
Oceania	1	5.3
**Sample size**		
<100	5	26.3
101-200	8	42.1
>200	6	31.6
**Gender**		
Only males	2	10.5
Only females	8	42.1
Both	9	47.4
**Evaluated the effect of PA during childhood/adolescence**		
Yes	16	84.2
No	3	15.8
**Evaluated the effect of PA during adulthood**		
Yes	16	84.2
No	3	15.8
**Measurement of outcomes**		
Only bone mineral content	3	15.8
Only bone mineral density	13	68.4
Both	3	15.8
**Evaluated the total body**		
Yes	5	26.3
No	14	73.7
**Evaluated the lumbar spine**		
Yes	15	79.0
No	4	21.0
**Evaluated the femoral neck**		
Yes	13	68.4
No	6	31.6

Table 2 shows other characteristics of the studies included in this present review. Although 19 articles were found in this present search, only 11 different samples were studied. For example, 5 of the manuscripts included in this review were written using data from Amsterdam Growth and Health Longitudinal Study (AGAHLS) [43-47]. Twelve of the studies starts when subjects were up to 15 years-old [43,45-55] and the mean of time between first measurement of physical activity and measurement of considered outcome was 14.1 years (sd = 6.2 years). Different questionnaires to assess physical activity were used in these studies. Although the most part of these questionnaires were created by researchers themselves, other known questionnaires such as Baecke, Physical Activity Questionnaire (PAQ) and Kaiser Physical Activity Survey (KPAS) were also used [48,49,51,52,56]. About temporality of the information, two studies estimated physical activity during adolescence using retrospective questionnaire [57,58].

**Table 2:** Summary of the articles included in the present review

**References Country**	**Study**	**Sample size Sex**	**Age at baseline considered in the analysis**	**Follow-up duration**	**PA during adolescence**	**PA during early adulthood**	**Outcomes**	**Main results**
Bakker, 2003 [44] Netherlands	Amsterdam Growth and Health Longitudinal Study (AGAHLS)	466 Both	27 years	10 years	-	Questionnaire developed for the AGAHLS. Semi-structured. Previous 3 months. Evaluation by metabolic activity score per week (METPA) and mechanical activity score (MECHPA) evaluated by sum of ground reaction force of each PA reported.	BMD	MECHPA was positively associated with BMD in males (β = 0,090, p < 0,001). Subjects of both sex in 2^nd^ and 3^rd^ quartile of METPA had greater BMD.
							LS	
Barnekow-Bergkvist, 2006 [57] Sweden	-	36 Females	15-17 years	20 years	Questions on participation in PA: leisure-time sports activity (yes/no), membership of a sports club (yes/no), and kind of activity/ies.	Questions about leisure-time PA were collected regarding type of activity and frequency of overall PA. Only weight-bearing activities were taken into account.	BMD	Girls who were members of a sports club showed higher adult BMD in all sites. There was no association between current weight-bearing PA and adult BMD.
							TB	
							LS	
							FN	
Baxter-Jones, 2008 [48] Canada	Saskatchewan Pediatric Bone Mineral Accrual Study (PBMAS)	154 Both	8-15 years	15 years	PAQ-C was used on children and PAQ-A on adolescents many times. Nine items scored on a five-point Likert-type scale. Age and sex-specific Z-score was determined. Individuals were ranked into quartiles according to Z-score: highest - active, middle two quartiles- average, lowest – inactive.	PAQ-AD. Used only for controlling the effect of past PA on BMC.	BMC	Active males during childhood/adolescence had higher BMD at TB and FN than inactive ones and active females had higher BMD at FN than inactive.
							TB	
							LS	
							FN	

Cooper, 1995 [58] England	-	153 Females	-	21 years	Participation in sports at school asked in adulthood. Classified in ≤2 h and 2 or more hours per week.	Duration of outdoor walking (none, 1–30, 31–60, 61–120 and >120 min/day), participation in sports and PA at work.	BMC	More than 2 hours/week of participation in sports at school was associated with higher BMD at FN. Duration of walking was positively associated with higher LS and FN BMD.
							BMD	
							BMAD	
							LS	
							FN	
Delvaux, 2001 [49] Belgium	Leuven Longitudinal Study on Lifestyle, Fitness and Health (LLSLFH)	126 Males	13 years	27 years	A standardized questionnaire was used. Sport activities during the past year were registered. From the reported time and frequency of sport participation, a global average score of hours per week was calculated. No distinction was made between weight-bearing activities and others.	The same questionnaire used on adolescence. Baecke questionnaire. Four indices were calculated: PA at work, sports activities during leisure-time, PA during leisure time excluding sports, and the total PA index as the sum of the three previous indices.	BMC	Sports at 13y were not associated with bone mass. Sports at 18y were positively associated with LS BMC. Occupational and leisure-time PA (excluding sports) were not related to bone mass. Baecke sports index was positively associated with TB BMD and LS BMC and BMD.
							BMD	
							TB	
							LS	
Groothausen, 1997 [43] Netherlands	Amsterdam Growth and Health Longitudinal Study (AGAHLS)	182 Both	13 years	14 years	Questionnaire developed for the AGAHLS. Semi-structured. Previous 3 months. PS determined from 0 to 3 according to ground reaction force of each PA. Two different PS scores: A – sum of all PS, B – the highest PS.	Same procedures used during adolescence period. PA evaluated at 21 and 27 years.	BMD	PS evaluated by sum of all PS in all periods (13–16 years;13–21 years; 21–27 years and whole period) was associated with LS BMD. PS evaluated by the highest PS was associated with LS BMD in 3 periods (13-21 years; 21–27 years and whole period).
					PA evaluated from 13 to 16 years.			
							LS	
Kemper, 2000 [46] Netherlands	Amsterdam Growth and Health Longitudinal Study (AGAHLS)	182 Both	13 years	16 years	Questionnaire developed for the AGAHLS. Semi-structured. Evaluation by metabolic activity score per week (METPA) and mechanical activity score (MECHPA) evaluated by sum of ground reaction force of each PA. PA evaluated from 13 to 16 years.	Same procedures used during adolescence period. PA evaluated at 21 and 27 years.	BMD	Positive association was found between METPA from 13 to 16 y and LS BMD only in males. MECHPA in young adulthood was associated with LS BMD in both sexes. No association was found between METPA and FN BMD. Positive association was found between MECHPA and FN BMD, adjusted for gender
							LS	
							FN	
Kemper, 2002 [45] Netherlands	Amsterdam Growth and Health Longitudinal Study (AGAHLS)	302 Both	13 years	19 years	Questionnaire developed for the AGAHLS. Semi-structured. Evaluation by metabolic activity score per week (METPA) and mechanical activity score (MECHPA) evaluated by sum of ground reaction force of each PA. PA evaluated from 13 to 16 years.	Same procedures used during adolescence period. PA evaluated at 21, 27, 29 and 32 years. Only measures performed on 21 and 27 years were analyzed.	BMD	METPA and MECHPA scores from total period (13-27y) were positively associated with LS BMD. METPA in the teenage period (13-16y) and MECHPA in young adulthood period (21-27y) were also positively associated with LS BMD.
							LS	
Lloyd, 2004 [50] United States	Penn State Young Women’s Health Study	75 Females	12 years	10 years	Questionnaire based on existing instruments. The questionnaire listed 28 activities, including school-based activities; outside-of-school organized activities; and individual activities. The cumulative sports exercise score was an arithmetic sum. Questionnaire applied at least once per year from 12 to 18y.	Same procedures used during adolescence period. Questionnaire applied at least once per year up to 22 years.	BMD	The cumulative sports-exercise score was positively correlated to FN BMD.
							FN	
McGuigan, 2002 [51] Ireland	Young Hearts Project	460 Both	12-15 years	10 years	PA scores in adolescence were calculated according to a method which assessed normal daily activity patterns based around the typical school day. Activities were scored from 1–100 according to their frequency, intensity and duration.	Modification of the Baecke questionnaire, which records work-related PA, sports-related PA, and non-sports leisure activity. A total activity score was obtained from the sum of scores in these domains to give a total score ranging from 3 to 15.	BMD	Exercise history was the most important predictor of LS BMD in men. PA was also the strongest predictor of FN BMD in men. The results were almost identical when using exercise data collected at the age of 12–15 years.
							LS	
							FN	
Mein, 2004 [56] Australia	-	62 Females	18.5 years	9 years	Physical activity questionnaire (PAQ) was used. It measured additional sporting pursuits. To compare the two questionnaires, the units of the PAQ scores were transformed by adding the product of the z-score of the PAQ and the SD of KPAS (sports and exercise index) to this mean of KPAS.	Kaiser Physical Activity Survey (KPAS) was used to evaluate habitual PA and exercise. Four indices could be calculated—Domestic, Occupational, Active Living, and Sports and Exercise. The average of these scores was expressed as a summary score.	BMD	Average PA was positively correlated with LS and FN BMD.
							LS	
							FN	
Neville, 2002 [52] Ireland	Young Hearts Project	443 Both	12-15 years	8-10 years	PA scores in adolescence were calculated according to a method which assessed normal daily activity patterns based around the typical school day. Activities were scored from 1–100 according to their frequency, intensity and duration.	Modification of the Baecke questionnaire, which records work-related PA, sports-related PA, and non-sports leisure activity.	BMD	In males, PA during adolescence was associated only with FN BMD. PA on young adulthood was associated with both LS and FN BMD in males. In females, the PA in both periods was not associated with BMD.
							LS	
							FN	
						A total activity score was obtained from the sum of scores in these domains to give a total score ranging from 3 to 15.		
Petit, 2004 [53] United States	Penn State Young Women’s Health Study	76 Females	12 years	10 years	Questionnaire based on existing instruments used from 12 to 18y. The questionnaire listed 28 activities, including school-based activities; outside-of-school organized activities; and individual activities. Cumulative sports exercise score was an arithmetic sum.	-	BMD	Sports exercise score during adolescence was not associated with FN BMD at 22 years and with change on BMD from 17 to 22 years.
							FN	
Uusi-Rasi, 2002 [60] Finland	-	92 Females	25-30 years	4.2 years	-	PA was classified into 4 categories according to type and frequency: (1) ‘high’ vigorous PA ≥ 2 times a week, (2) ‘moderate’ vigorous PA ≤ once a week or less demanding PA few times a week, (3) ‘low’ less demanding PA once a week or very light PA several times a week (4) ‘no activity’. Category 1 was the PA + group and categories 3 and 4 were PA– groups.	BMC	There were no statistically significant differences for the FN BMC between the PA + and PA– groups
							FN	
Uusi-Rasi, 2008 [59] Finland	-	133 Females	25-30 years	10 years	-	PA was classified into 4 categories according to type and frequency: (1) ‘high’ vigorous PA ≥ 2 times a week, (2) ‘moderate’ vigorous PA ≤ once a week or less demanding PA few times a week, (3) ‘low’ less demanding PA once a week or very light PA several times a week (4) ‘no activity’. Category 1 was the PA + group and categories 3 and 4 were PA– groups.	BMC	There was no statistical difference between PA + and PA- group at FN BMC evaluated by three repeated measures (baseline, 5-year, 10-year).
							FN	
Valimaki, 1994 [61] Finland	Young Finns study	264 Both	9-18 years	11 years	Subjects were asked about weekly frequency of PA exceeding 30 minutes per performance. This same question was used in baseline and 6-year follow-up. Having two or more weekly sessions was called 1 and less than two sessions was called 0. PA on childhood or adolescence was analyzed with PA on adulthood.	Same question was used in 10-year follow-up. The sum of the three years answers ranging from 0 to 3 was calculated.	BMD	LS BMD was greater in males with PA evaluated as score 3. FN BMD was greater in both males and females with PA evaluated as score 3 than subjects with other PA values.
							LS	
							FN	
Van Langendonck, 2003 [54] Belgium	Leuven Longitudinal Study of Lifestyle, Fitness and Health (LLSLFH)	154 Males	13 years	27 years	Sports participation inventory was used. Information about the types of sports and the time per week was obtained. The score for 13–18y was calculated. Other two different analyses of PA were performed. 1) PS determined from 0 to 3 according to ground reaction force of each PA. Sum of all PS scores was calculated. 2) Groups were created from the ground reaction force: high, moderate, light or nonimpact.	Same questionnaire used during adolescence asked at 30, 35 and 40y. PS from adulthood was added to PS obtained on adolescence. Groups obtained on second analysis according to engagement on high (H) or nonimpact (N) in each period were: HH, HN, NN. NH group was excluded of analysis, as well as subjects whose sports participation did not meet these criteria.	BMD	PS score during adulthood was a positive predictor of TB and LS BMD. HH group showed greater LS BMD than HN and NN groups.
							TB	
							LS	
Wang, 2003 [55] United States	Berkeley Bone Health Study (BBHS)	341 Females	9-10 years	10-15 years	PA level assessed by self reported habitual activities, with scores derived by using MET values and time estimates (years 1, 3, and 5–10). Sedentary activity assessed by self-reports of weekly hours of television-video viewing (years 1, 3, and 5–10).	-	BMC	Physical activity was not associated with bone mass. Only sedentary activity on pre-puberty was negatively associated with FN BMD and BMAD.
							BMD	
							BMAD	
							TB	
							LS	
							FN	
Welten, 1994 [47] Netherlands	Amsterdam Growth and Health Longitudinal Study (AGAHLS)	182 Both	13 years	14 years	Questionnaire measuring habitual PA in the last 3 months. Only PA with a minimal of 4 METs were considered. The average weekly time spent in 3 activity level was collected: light (4-7METs), medium heavy (7-10METs) and heavy (>10METs). Total PA/week was the product of the time spent per level of intensity (1, 2 or 3). Only PA with a weight-bearing component was used. Adolescent period was considered from 13 to 17y	Same questionnaire used during adolescence. PA on young adulthood was analyzed on period between 13 and 22y and on total period – between 13 and 28y	BMD	Weight-bearing PA in all periods (13-17y; 13-22y and 13-27y) was positively associated with LS BMD only in males.
							LS	

*PA* Physical activity, *BMC* Bone mineral content, *BMD* Bone mineral density, *BMAD* Bone mineral apparent density, *TB* Total body, *LS* Lumbar spine, *FN* Femoral neck, *PS* Peak strain.

Three studies considered only weight-bearing physical activities in the analysis [47,49,57], while the rest used general physical activity. The nineteen included studies showed twelve different ways to classify general physical activity by questionnaires. They used the following: a standard value for groups of activities according to intensity, times the resting metabolic rate (RMR) x minutes per week [44]; physical activity at least once per week (yes/no) [57]; membership of a sports club (yes/no) [57]; scores using different ranges of values [48-53,56]; categories of outdoor walking [58]; participation in sports at school for at least 2 hours (yes/no) [58]; hours of sports activity per week [49,54]; number of metabolic equivalents (METs) per week [46]; MET score in levels determined according to intensity of each activity multiplied by the measured duration in minutes [45,47]; four categories of physical activity, the first category was the active group and the third and fourth categories were the inactive group [59,60]; individuals who performed two or more sessions of physical activity exceeding 30 minutes per performance were considered active (1) and inactive (0) for the others – subjects had the sum of the three years' answers ranging from 0 to 3 for physical activity from adolescence to adulthood [61] and; MET-times per week – annual average of metabolic equivalent for each activity multiplied by weekly frequency [55].

Physical activity was also analyzed using peak strain scores created by Groothausen [43]. Five manuscripts used this score [43-46,54], whereas four out of these manuscripts were conducted with AGAHLS sample. Peak strain score consists of evaluation of physical activity based on ground reaction forces of different physical activities. Activities with ground reaction force less than 1 time the body weight such as cycling and swimming have the peak score 0, activities with peak score between 1 and 2 times the body weight – weight bearing activities such as jogging, walking and ballroom dancing – have the peak score 1, activities with ground reaction force between 2 and 4 times the body weight – activities including sprinting and turning actions such as tennis, aerobics and soccer have the peak score 2, activities including jumping actions with ground reaction force greater than 4 times the body weight such as basketball and gymnastics have the peak score 3. Peak strain score may be used in two ways. Firstly, the peak scores of each activity are added up to others. Second option consists in selecting only the highest peak scores [43]. The evaluation in these studies was performed independent of frequency and duration of activities.

### Quality assessment

Concerning quality assessment, results of evaluation criteria adapted from Downs & Black [10] are shown in Table 3. Studies could reach the maximum of 24 points, divided into 5 different aspects – reporting, external validity, bias, confounding and power. No study reached this limit. Scores were on average 16.6 points (SD = 3.0). The lowest score was 14 points [43,48,49,58], whereas only one study reached the highest score of 20 points [52]. Concerning questions about reporting, only 2 manuscripts had maximum score of 10 points [51,57]. The main problem in this sub-scale was the lack of studies reporting the characteristics of patients lost to follow-up. Only 7 studies reported no difference between followed-up subjects and those who dropped out [45,46,51-53,56,57]. Regarding sub-scale of external validity, around half of the manuscripts did not report at least one out of the two questions about representativity of the recruited sample at the baseline and about representativity of the followed-up subjects. More frequent fragility of all studies in sub-scale of bias was no attempting to blind the subjects and those who were measuring the outcomes to the exposures. No study reported these questions in the methods section. Concerning sub-scale of confounding, few studies took into account the losses of subjects to follow-up. On the other hand, all studies recruited the subjects of different grades of physical activity from the same population. No study reported sample size calculation, sufficient power to detect an important difference or minimum detectable difference on values of bone mass between grades of physical activity.

**Table 3:** Evaluation criteria adapted from Downs & Black (1998)

**Studies**	**Reporting**	**External validity**	**Bias**	**Confounding**	**Power**	**Overall**
	**0–10**	**0–2**	**0–7**	**0–4**	**0–1**	**0–24**
Bakker et al. (2003)	7	2	5	3	0	17
Barnekow-Bergkvist et al. (2006)	10	1	5	3	0	19
Baxter-Jones et al. (2008)	7	0	5	2	0	14
Cooper et al. (1995)	6	2	4	2	0	14
Delvaux et al. (2001)	7	0	5	2	0	14
Groothausen et al. (1997)	7	0	5	2	0	14
Kemper et al. (2000)	8	2	5	3	0	18
Kemper et al. (2002)	5	2	5	4	0	16
Lloyd et al. (2004)	7	2	5	3	0	17
McGuigan et al. (2002)	10	1	5	3	0	19
Mein et al. (2004)	9	0	5	3	0	17
Neville et al. (2002)	9	2	5	4	0	20
Petit et al. (2004)	9	2	5	3	0	19
Uusi-Rasi et al. (2002)	8	0	5	3	0	16
Uusi-Rasi et al. (2008)	8	0	5	3	0	16
Valimaki et al. (1994)	9	2	5	3	0	19
Van Langendonk et al. (2003)	9	2	5	3	0	19
Wang et al. (2003)	8	2	5	3	0	18
Welten et al. (1994)	8	0	5	3	0	16
**Mean (SD)**	7.9 (1.3)	1.2 (1.0)	4.9 (0.2)	2.9 (0.6)	0.0 (0.0)	16.6 (3.0)

### Findings according to anatomical site

Findings in this section were summarized by analyses results. More details are presented in the Additional file 1.

#### Total body bone mineral content and density

Five studies included in this review evaluated association between physical activity and total body bone mineral content or density [48,49,54,55,57]. Concerning 9 analyses using physical activity during adolescence (6 in females), only two out of these showed positive association between physical activity and total body BMD or BMC [48,57]. Respecting analysis performed using physical activity on adulthood (5 analyses – 4 in males), only two analyses were positively associated with bone mass [49,54]. The only analysis that used cumulative physical activity did not show positive association with bone mineral density in males [54].

#### Femoral neck bone mineral content and density

Regarding thirteen manuscripts included in this review which evaluated association between physical activity and measurements of femoral neck bone mass, they showed 29 different analyses [46,48,50-53,55-61]. Concerning the 14 analyses using the exposure only during adolescence, 6 found positive association between physical activity and bone mineral density or content [48,51,52,57,58], in addition one analysis showed negative association between sedentary behavior (hours of television-video viewing) and bone density [55]. Regarding analyses using physical activity in adulthood (11 analyses), only 4 analyses were positively associated with these bone outcomes [46,51,52,58]. Among studies that evaluated cumulative physical activity from adolescence to adulthood, the four performed analyses were all positively associated.

#### Lumbar spine bone mineral content and density

Fifteen studies reported findings of association between physical activity measurements and bone mineral density or content [43-49,51,52,54-58,61]. They showed 52 different analyses between exposure of interest and outcome. Concerning the 22 analyses that used physical activity during adolescence, only 7 out of these found positive association with bone mineral density or content [43,45-47,49,51,57], whereas 11 analyses out of 21 carried out using physical activity in adulthood were positively associated [43-46,49,51,52,54,58]. Only 2 analyses, performed with females, did not find association between cumulative physical activity from adolescence to adulthood and bone mineral density or content (overall = 9) [47,61].

### Findings according to sex and physical activity measurement

**Figure 2:**
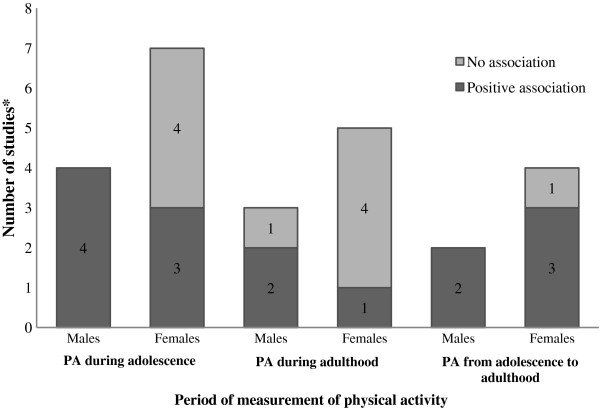
**Number of studies according to the association between general physical activity and bone mass by sex and period of physical activity measurement.** Criterion for positive association was the presence of at least one positive association between physical activity with at least one anatomical site (total body, lumbar spine or femoral neck). ^*^Only first published manuscripts with the studied samples were included.

Figure 2 shows the number of studies with at least one positive association between general physical activity and bone mineral content or density according to the period of physical activity measurement and sex. In this figure were included only first published manuscripts using each studied sample and each period of assessment of physical activity, to avoid possible biased conclusions caused by inclusion of more than one study that used the same sample. Out of 18 manuscripts included in this review, thirteen manuscripts are shown in Figure 2. Concerning manuscripts that evaluated these associations in males, all studies that performed association between general physical activity during adolescence and bone mineral density or content on young adulthood found at least one positive association with at least one anatomical site [47-49,52]. There seems to be no consensus on literature about existence of positive or absent association between general physical activity on young adulthood and bone mass at same period of life [46,49,52]. In contrast, regarding general physical activity from adolescence to adulthood, the only two existing studies showed positive association with bone mineral density or content in young adulthood [47,61].

In females, there seems to be no consensus or lack of association between physical activity during adolescence and bone mass in young adulthood, since more studies reported absence than positive associations [47,48,52,53,55,57,58]. Furthermore, the findings of studies carried out with females showed that there was no association between general physical activity during adulthood and bone mass measurements [26,46,57,58,60]. However, the majority (*n* = 4) of the studies that evaluated association between cumulative general physical activity from adolescence to adulthood and bone mineral density or content in young adulthood showed positive associations [47,50,56,61].

Concerning the only two studied samples (AGAHLS and LLSLFH) [43-46,54] in which peak strain scores were used to evaluate physical activity in addition to general physical activity, it seems that physical activity evaluated by peak score showed more positive associations with bone mass than general physical activity. Moreover, analyses performed with peak score in adulthood were more positively associated with bone mass than analyses using the adolescence period. Since one study [54] was carried out only with males, it is impossible to make pooled conclusions concerning differences in effect of peak score by gender.

## Discussion

Nineteen manuscripts met inclusion criteria. Lumbar spine was the skeletal site most studied (n = 15). Different questionnaires were used for physical activity evaluation. Peak strain score was also used to evaluate physical activity in 5 manuscripts. Lack of statistical power calculation was the main problem found in the quality assessment of all studies. More positive associations between physical activity and bone mass were found in males than in females and when physical activity measurements were done from adolescence to adulthood – than when evaluated in only one period.

This is the first study to systematically review the literature about cohort studies that evaluated the effect of physical activity on bone mass measurements in young adults. The choice of this age group was based on the scarcity in the literature about this subject in individuals who are in the maintenance phase of bone mass, since several studies performed in adults are in pre or menopausal women and older people, who have an increased risk for hip fracture. The main strength of this study was the selection of articles performed independently by two reviewers and a third revision in case of disagreement. This strategy reduces the possibility that some important article might not be identified. Another positive aspect of this study was the quality assessment of these papers which helps to detect fragilities of each included study.

We found 11 different samples with prospective physical activity evaluation and bone mass measurements in young adulthood. It was difficult to summarize findings from the 19 included studies, since there is much heterogeneity among them. The sources of heterogeneity were anatomical sites of bone mass measurements, the evaluated genders and mainly different physical activity assessments.

The quality assessment by Downs & Black criterion [10] showed that the most important aspect found was the lack of statistical power analysis, since no manuscripts reported the power calculation. In addition, as the most part of the studies had a sample size lower than 200 subjects, it is possible that some analyses were not statistically significant due to their low statistical power. Another important aspect in the quality assessment was that the characteristics of the losses were not described in some manuscripts. On the other hand, all studies included at this review used in the analysis adjustment at least for the body size, evidencing the authors’ concern with the statistical analyses.

Findings from the studies included showed that around half of the analyses using lumbar spine or femoral neck bone mass as outcome were positively significant, whereas only one third of them were positively significant for total body bone mass. One explanation for this fact is that bone adaptation is limited to loaded regions [5]. Other reason could be the fact that the total body site also includes no weight bearing anatomical sites, such as the wrist, and the majority of physical activities practiced by healthy individuals are weight bearing (walking, running, etc.) and specific activities such as handball and weight lifting are less practiced [47]. These arguments could explain why higher percentages of these analyses were positively associated with weight bearing sites (lumbar spine and femoral neck) than with total body.

The bone mass peak is prior to age 20 years at the proximal femoral sites and 6 to 10 years later for total skeletal mass [3]. So, it would be expected that a higher number of positively associations were found for analyses using physical activity during adolescence as exposure, life period with higher linear growth. However, lower percentages of positive associations with bone mass measurements were found for physical activity only during adolescence (around one third) and only in young adulthood (almost half) than for physical activity from adolescence to adulthood (around 80%).

Sports practice during adolescence are related to higher physical activity levels in adulthood, so that associations found between physical activity in young adulthood and bone mass could reflect sports activities in the past, which have greater ground reaction force and, therefore, are more osteogenic [52]. This fact would explain why almost all analyses between bone mass and physical activity considering the whole period of adolescence and adulthood were positively associated.

The adjustment for confounders is other aspect that should be appointed. Due to the fact that the body size is highly correlated to bone mass, all studies included weight and/or height or body mass index in the multivariate analyses. Most studies showed only coefficients of linear regressions with adjustment for body size. Thus, it is difficult to know the real differences introduced by the body size. However, the effect of body size could reduce the coefficient of the association between physical activity and bone mass, as observed in study with AGAHLS sample [46]. Calcium intake was not included as confounder in only one manuscript [43]. Other nutritional variables, such as energy intake and phosphorus, protein, carbohydrates, fat, magnesium and alcohol were included in the models of some studies [48,49,51-54,56,58]. Fewer studies considered smoking in their analyses [48,49,51,52,57,61]. Moreover, reproductive factors, such as parity, breastfeeding and time from weaning were included in multivariate analyses of few studies [48,56,59,60]. Since several differences in the statistical tests and adjustment strategies were found in these studies, it is difficult to determine the magnitude of bias that could be introduced by these differences. However, studies about this subject should carefully take into account the whole hierarchical model and its factors in order to avoid biased results.

Although only around one third of analyses between physical activity during adolescence and bone mass measurements were positively associated, when the results by sex are showed, important differences between genders are observed, since the most part of associations were found in males. The lack of association in females, besides biological differences, could be explained by their lower participation in sports and vigorous activities or an insufficient physical activity level to create a demonstrable effect on their bone mass [26,52]. Thus, though participation in moderate activities as walking is not different between genders, in the worldwide context males are more likely to participate in vigorous-intensity physical activity than are females [62].

In addition, considering differences on effect of physical activity during adolescence on bone mass between genders, it has been suggested that boys’ bones are more sensitive to loading than girls’ bones [63]. Moreover, it seems that the effect of physical activity on bone status reduces in females, but not in males [64]. However, the most important explanation for lack of association between physical activity and bone mass in females is their less frequency in sports involving high peak strain and ground reaction force enough to increase their bone mass [26].

From studies included in this review, it is impossible to recommend the amount of physical activity necessary to promote benefits on bone health, since different instruments for physical activity evaluation were used in these studies. In addition, it is impossible to determine the pooled magnitude of effect of physical activity in each age on bone mass due to the same reason. The current guidelines themselves did not report a consistent recommendation for enough physical activity to improve the bone health. Recommendations for children and adolescents only appoint that it is important to spend a percentage of 60 minutes of daily physical activity in bone-strengthening activities on at least 3 days a week. For adults there is no specific recommendation to promote bone health [65].

It seems to be a consensus that high impact sports are the main activities that maximize bone mass accumulation and maintenance and also reduce the loss of bone mass on elderly and postmenopausal period. However, it is not clear which is the best training method for enhancing bone mass, though scientific evidence points to a combination of high impact exercises with weight-lifting exercises [5]. The studies included in this review did not compare the effect between different activities, but in sample from AGAHLS, associations between physical activity and bone mineral density in lumbar spine and femoral neck in different times of evaluation of both exposure and outcomes were more consistent using peak strain score than when general physical activity was used. This strengthens that current recommendations of physical activity, mainly for adults, may not be adequate to attend the needs of bone health.

Besides type of activities, other difficult questions to be responded by the literature are concerned with how many sessions (frequency) and how long (duration) is needed to cause bone adaptation. Such studies did not respond these questions, but several randomized studies with positive results have used 2–3 training days per week, though this depends on the type of activity practiced [5].

The pooled findings show that more studies with positive associations between physical activity and bone mass were seen in males than females. The relationship of physical activity only during adolescence or adulthood and bone mass was not found in young women, mostly likely because they did not participate in peak strain activity on a sufficiently frequent basis. Moreover, analyses performed for each period did not discard the effect of physical activity posteriorly or previously and the tracking effect should be considered in this question since people who were highly active in adolescence are more likely to be active in adulthood. In addition to results found in females, since physical activity only during adolescence or adulthood seems to have no effect on bone mass, it is important to promote physical activity in both growth and maintenance periods for them due to the fact that women with more engagement in physical activity in the whole period from adolescence to adulthood may have benefits to bone health as well as males too.

The physical activity during the growth period seems to be highly important for males taking into account the positive effect on total period – from adolescence to adulthood and the maintaining across the lifespan. However, recent publication appointed that few data available indicate that exercise benefits in bone mineral density are eroded in the long term, indicating that residual factors caused by physical activity in the growth period such as structural changes, muscle strength, coordination and balance could be more important to prevent fractures in later life [66].

## Conclusions

Findings from these studies show no consensus, but it seems that promoting sports involving high peak strain (e.g., team sports) among growth and young adulthood period would result in improvements in peak bone density. Therefore, sports promotion in public places such as schools is important to provide opportunities for physical activity for the population. There is also the need of promoting vigorous-intensity physical activity especially in the female group, since besides lower bone mass explained by hormonal differences between genders, physical activity may play an important role on reducing the risk of osteoporosis in women. A challenge for studies in the field of physical activity and health is to encourage the use of standard instruments and analysis strategies which enable more comparison between studies and pooled conclusions. Moreover, there is the need of birth cohort studies showing results of the effect on bone mass of physical activity since childhood, in addition to the need of carrying out studies in low and middle-income countries where activity patterns and ethnicity are different from the high income countries.

## Competing interest

The authors declare that there are no conflicts of interest.

## Authors' contributions

RMB conceptualized the study, carried out the selection of the manuscripts, the quality assessment and wrote the text. JMM carried out the selection of the manuscripts and the quality assessment. DPP coordinated the study, carried out the selection of the manuscripts, contributed to the writing and revision of the manuscript. All authors read and approved the final manuscript.

## Pre-publication history

The pre-publication history for this paper can be accessed here:

http://www.biomedcentral.com/1471-2474/14/77/prepub

## Supplementary Material

Additional file 1 Associations (Yes/No) between physical activity and bone mass stratified by sex, anatomical site and age of physical activity assessment. Click here for file
