# Supplementary material for: Physical activity during life course and bone mass: a systematic review of methods and findings from cohort studies with young adults
Source: BMC Musculoskelet Disord. 2013 Mar 4;14:77. doi: 10.1186/1471-2474-14-77 (PMC3599107; doi:10.1186/1471-2474-14-77)
Supplement: Additional file 1 — Associations (Yes/No) between physical activity and bone mass stratified by sex, anatomical site and age of physical activity assessment. [file 1471-2474-14-77-S1.pdf]

Additional file 1. Associations (Yes/No) between physical activity and bone mass stratified by sex, anatomical site and age of physical activity assessment.

References	Males										Females									
	Sample	TB			FN			LS			Sample	TB			FN			LS		
		AD	AH	AD /AH	AD	AH	AD /AH	AD	AH	AD /AH		AD	AH	AD /AH	AD	AH	AD /AH	AD	AH	AD /AH
Bakker, 2003	225	-	-	-	-	-	-	-	YES[MC]<br>NO[S/MT]	-	241	-	-	-	-	-	-	-	NO[MC]	-
Barnekow-Bergkvist, 2006	-	-	-	-	-	-	-	-	-	-	36	YES	NO	-	YES	NO	-	YES	NO	-
Baxter-Jones, 2008	72	YES[C]	-	-	YES[C]	-	-	NO[C]	-	-	82	NO[C]	-	-	YES[C]	-	-	NO[C]	-	-
Cooper, 1995	-	-	-	-	-	-	-	-	-	-	153	-	-	-	NO[C]<br>YES	NO[C]<br>YES	-	NO[C]<br>NO	NO[C]<br>YES	-
Delvaux, 2001	126	NO[C]<br>NO	NO[C]<br>YES	-	-	-	-	YES[C]<br>NO	YES[C]<br>YES	-	-	-	-	-	-	-	-	-	-	-
Groothausen, 1997	182[S]	-	-	-	-	-	-	YES[S]	YES[S]	YES[S]	-	-	-	-	-	-	-	-	-	-
Kemper, 2000	182[S]	-	-	-	NO[S/MT]<br>NO[S/MC]	NO[S/MT]<br>YES[S/MC]	-	-	-	-	-	-	-	-	-	-	-	-	-	-
	84	-	-	-	-	-	-	YES[MT]<br>NO[MC]	NO[MT]<br>YES[MC]	-	98	-	-	-	-	-	-	NO[MT]<br>NO[MC]	NO[MT]<br>YES[MC]	-
Kemper, 2002	182[S]	-	-	-	-	-	-	YES[MT]<br>NO[MC]	NO[MT]<br>YES[MC]	YES[MT]<br>YES[MC]	-	-	-	-	-	-	-	-	-	-
Lloyd, 2004	-	-	-	-	-	-	-	-	-	-	75	-	-	-	-	-	YES	-	-	-
McGuigan, 2002	244	-	-	-	YES	YES	-	YES	YES	-	216	-	-	-	NO	NO	-	NO	NO	-
Mein, 2004	-	-	-	-	-	-	-	-	-	-	62	-	-	-	-	-	YES	-	-	YES
Neville, 2002	238	-	-	-	YES	YES	-	NO	YES	-	205	-	-	-	NO	NO	-	NO	NO	-
Petit, 2004	-	-	-	-	-	-	-	-	-	-	76	-	-	-	NO	-	-	-	-	-
Uusi-Rasi, 2002	-	-	-	-	-	-	-	-	-	-	92	-	-	-	-	NO[C]	-	-	-	-
Uusi-Rasi, 2008	-	-	-	-	-	-	-	-	-	-	133	-	-	-	-	NO[C]	-	-	-	-
Valimaki, 1994	111	-	-	-	-	-	YES	-	-	YES	153	-	-	-	-	-	YES	-	-	NO
Van Langendonck, 2003	154	-	NO[MT]<br>YES[MC]	NO[MC]	-	-	-	-	NO[MT]<br>YES[MC]	YES[MC]	-	-	-	-	-	-	-	-	-	-
Wang, 2003	-	-	-	-	-	-	-	-	-	-	341	NO[C]<br>NO[C/SD]<br>NO<br>NO[SD]	-	-	NO<br>YES[SD]	-	-	NO<br>NO[SD]	-	-
Welten, 1994	84	-	-	-	-	-	-	YES	-	YES	98	-	-	-	-	-	-	NO	-	NO

TB – Total body; FN – Femoral neck; LS – Lumbar Spine; AD – Adolescence; AH – Adulthood; AD/AH – From adolescence to adulthood; C – Bone mineral content; S – analysis performed with both sexes; MT – Physical activity evaluated by METs; MC – Physical activity evaluated by mechanical strain; SD – Sedentary activity . Results with no legends used bone mineral density as outcome.
